# Long-Term Effects of Transcranial Direct-Current Stimulation in Chronic Post-Stroke Aphasia: A Pilot Study

**DOI:** 10.3389/fnhum.2014.00785

**Published:** 2014-10-14

**Authors:** Lucilla Vestito, Sara Rosellini, Massimo Mantero, Fabio Bandini

**Affiliations:** ^1^Rinascita Vita, Genova, Italy; ^2^Department of Neurology, San Paolo Hospital, Savona, Italy

**Keywords:** tDCS, chronic aphasia, stroke, long term effects, brain stimulation

## Abstract

Transcranial direct-current stimulation (tDCS) has been suggested to improve language function in patients with post-stroke aphasia. Most studies on aphasic patients, however, were conducted with a very limited follow-up period, if any. In this pilot, single-blind study on chronic post-stroke aphasic patients, we aimed to verify whether or not tDCS is able to extend its beneficial effects for a longer period of time (21 weeks after the end of stimulation). Three aphasic patients underwent anodal tDCS (A-tDCS, 20 min, 1.5 mA) and sham stimulation (S-tDCS) over the left frontal (perilesional) region, coupled with a simultaneous naming training (on-line tDCS). Ten consecutive sessions (5 days per week for 2 weeks) were implemented. In the first five sessions, we used a list of 40 figures, while in the subsequent five sessions we utilized a second set of 40 figures differing in word difficulty. At the end of the stimulation period, we found a significant beneficial effect of A-tDCS (as compared to baseline and S-tDCS) in all our subjects, regardless of word difficulty, although with some inter-individual differences. In the follow-up period, the percentage of correct responses persisted significantly better until the 16th week, when an initial decline in naming performance was observed. Up to the 21st week, the number of correct responses, though no longer significant, was still above the baseline level. These results in a small group of aphasic patients suggest a long-term beneficial effect of on-line A-tDCS.

## Introduction

Non-invasive brain stimulation (NIBS) techniques have recently emerged in restorative neurology due to their hypothetical advantage in enhancing the efficacy of traditional therapeutic intervention (Holland and Crinion, 2012). In this view, the re-discovery of the application of a direct-current flow of low intensity (1–2 μA) has raised much interest. This technique is known as transcranial direct-current stimulation (tDCS). It acts by a tonic modulation of the resting membrane potential of the cortical neurons, which occurs in an opposite direction, depending on the polarity (anodal vs. cathodal) of the electrodes placed on the chosen areas. It is commonly stated that cathodal stimulation (C-tDCS) decreases cortical excitability due to neural hyperpolarization, while anodal stimulation (A-tDCS) reaches the opposite effect by a subthreshold depolarization (Nitsche and Paulus, 2000).

Recent evidence suggests that tDCS is a safe and (relatively) painless instrument for manipulating performance in a variety of motor and cognitive domains, and investigators have started exploring the use of tDCS as a possible rehabilitative tool for patients with post-stroke deficits, including impairment of language. These works converged in a body of evidence that A-tDCS can improve language performances when applied to the left hemisphere (LH), particularly on the frontal cortex (Monti et al., 2013), whose residual activity is supposed to allow speech production (Fridriksson et al., 2010).

Most of the studies conducted on aphasic patients utilized an on-line approach, i.e., stimulating the damaged areas while the patient is undergoing specific language rehabilitation training, with the aim of generating synergistic effects. In this way, modification in cerebral plasticity might be better achieved by targeting specific pools of neurons (Bolognini et al., 2009). On the other hand, the two off-line studies conducted so far, administering either single (Monti et al., 2008) or repetitive (Volpato et al., 2013) sessions of tDCS when patients were at rest, obtained conflicting results.

The long-term effects of tDCS are largely unknown. As compared to the other method of NIBS (repetitive transcranial magnetic stimulation or rTMS), tDCS seems to produce longer effect on neural excitability (Paulus, 2003) and, even if rTMS and tDCS are not exchangeable modalities, one might hypothesize that tDCS is capable of producing long-lasting effects as well. Surprisingly, despite there is some evidence for a potentially cumulative effect of tDCS in improving motor recovery in patients with stroke (Boggio et al., 2007; Khedr et al., 2013) and motor learning in healthy subjects (Reis et al., 2009), tDCS studies to date on aphasic patients were not aimed to explore the duration of its efficacy. The longest follow-up data available in aphasic patients was 3 weeks post-training (Fiori et al., 2011; Fridriksson et al., 2011).

In this pilot study, we implemented 10 tDCS (sham and anodal) consecutive sessions (5 days per week over a 2-week period), in three chronic post-stroke aphasic patients, coupled with a simultaneous picture-naming training. Two sets of figures differing for difficulty in terms of frequency were used (one set for each 5-day intervention). Our hypothesis was that repeated sessions of on-line tDCS could lead to a long-lasting improvement in language function. Thus, we wanted to verify whether or not such a technique is able to extend its beneficial effects for a longer follow-up period (21 weeks after the end of stimulation).

## Materials and Methods

Three patients with chronic aphasia were enrolled in the study: two were males and one female. Inclusion criteria were right-handedness, single LH damage, more than 1 year after stroke onset, native Italian language. Exclusion criteria were sensitive scalp, presence of intracranial metal implants, and history of epilepsy.

**Figure 1:**
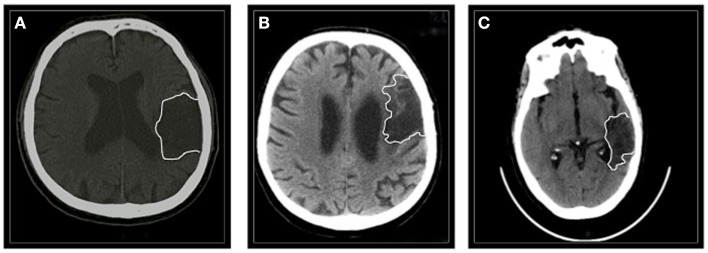
**The figure shows a single CT scan acquisition of all the three patients**. **(A)** Patient #1 suffered from a left (fronto)temporal hemorrhage. **(B)** Patient #2 had a left frontal ischemic stroke. **(C)** Patient #3 had a left temporal hemorrhage.

Patients #1 and #3 had suffered from a hemorrhagic lesion involving the left (fronto)temporal region (time after stroke 20 and 26 months, respectively); patient #2 had an ischemic stroke involving the left frontal area (time after stroke 64 months) (Figure 1).

All three patients had been following a standard language rehabilitation program (picture and verbal description naming) for at least 1 year. The aphasic disorder was assessed 1 week before the stimulation using two standardized language tests, namely the Aachener Aphasie Test (AAT) (Luzzati et al., 1991) and the Boston Naming Test (BNT) (Kaplan et al., 1983). Cut-off values for AAT naming impairment were as follows: 0–42 very high; 43–58 high; 59–70 medium; above 70 minimal or absent. Cut-off value for BNT expressed for age was 50/60. According to AAT, patients #1 and #2 were classified as having a non-fluent type of aphasia, with a high severity for the patient #1 and a very high severity for patient #2. Patient #3 was diagnosed as an anomic aphasic of moderate severity. Written and oral comprehension was well preserved (Table 1).

**Table 1:** **Demographic and clinical data of the three aphasic patients**.

Patient	Sex	Age	Post-stroke onset (months)	Lesion location	AAT naming	AAT comprehension (oral–written)	BNT
1	M	62	20	Left (F)T hemorrhage	57/120	110/120	20/60
2	M	65	64	Left F infarct	27/120	106/120	11/60
3	F	67	26	Left T hemorrhage	83/120	119/120	22/60

An informed consensus was obtained from participants prior to the beginning of the experiment.

The picture-naming treatment utilized two different sets of 40 black and white three-dimensional figures, consisting of objects belonging to living and non-living semantic categories.

The first set of figures included 15 high frequency [i.e., macchina (car), fiore (flower)] and 15 low-frequency objects [i.e., mappamondo (globe), cavalluccio marino (seahorse)]. The second set included 10 high frequency [i.e., porta (door), pollo (chicken)] and 20 low-frequency objects [i.e., grattacielo (skyscraper), amaca (hammock)]. Ten high frequency verbs were included in the first set [i.e., dormire (to sleep), bere (to drink)], while 10 low-frequency verbs [i.e., applaudire (to clap), versare (to pour)] were present in the second set. The two figure sets were matched for semantic content and length (number of syllables per word) but not for word frequency. The second set was chosen in order to verify whether or not the difficulty in naming task could have a detrimental effect on the patients’ performance. The naming training with the first set of figures was administered daily for five consecutive days. The second naming training (using the second set of figures) was again given daily for five consecutive days, after a 2-day rest interval. The 40 figures of each set were randomly presented by the examiner (seated in front of the patient), simultaneously with S-tDCS and A-tDCS. Each stimulus lasted 25 s, with 5 s of interval between figures. The indication “action” was verbally given immediately before the verb presentation. Patients were asked to accurately name the figures and no phonemic cues were provided. In case of anomia, the correct name was not given. Accuracy was evaluated giving one point to each correct response.

**Figure 2:**
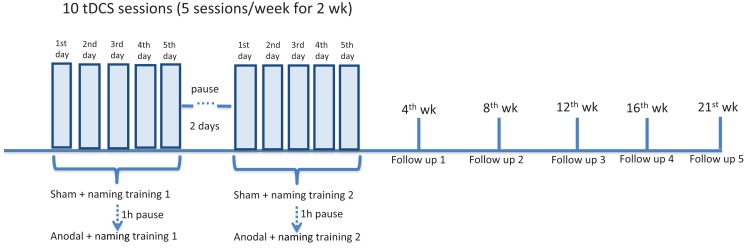
**Overview of experimental design**. All three patients underwent a daily naming training (first list of 40 figures) for five consecutive days. Concurrently, tDCS (20 min) was administered over the left frontal region. Two stimulations were given: first sham, then anodal, with a 60-min pause interval. After a 2-day pause interval, all the patients underwent a second daily naming training with a second list of 40 figures (more difficult in terms of word frequency). The same simultaneous tDCS paradigm was applied. At the beginning of each week (to measure baseline performance), at the end of the stimulation period, after 4, 8, 12, 16, and 21 weeks (first, second, third, fourth, and fifth follow-up), patients were shown the two lists of figures and asked to name them. The Aachener Aphasia Test (AAT) and the Boston Naming Test (BNT) were administered 1 week before the experiment, at the end of each stimulation period and at each follow-up (not shown in the figure).

The same tasks, including AAT and BNT, were performed at the end of each stimulation period. In the follow-up period, patients were again tested at week 4, 8, 12, 16, 21 after the end of each stimulation period (first and second set of figures were tested 1 week apart) (overview of the experimental design in Figure 2).

Stimulation with tDCS was delivered by a battery-driven, constant current simulator (Newronika srl, Italy), made of an LCD touch screen (HDC progr), a portable simulator (HDC stim), two holding bags of plant cellulose (5 cm × 5 cm), and two electrodes of conductive silicone. The electrodes were placed by means of a cap on the scalp overlying the left frontal (perilesional) site (active electrode), over the crossing point between T3-Fz and F7-Cz, according to the international 10–20 EEG system. The reference electrode was located over the contralateral supraorbital region. An electroconductive gel was applied under the electrodes to reduce contact impedance. Impedance was kept constantly below 5 kΩ. The study was a single-blind experiment. As such, the patients did not recognize the type of stimulation, while the examiner knew it. During the training task, A-tDCS (current of 1.5 mA) was delivered for 20 min. Current density (0.06 mA/cm^2^) was maintained below the safety limits (Poreisz et al., 2007). In the S-tDCS session (lasting 20 min as well), the current was turned off 30 s after the beginning of the stimulation and turned on for the last 30 s. In this way, the patients felt the itching sensation below the electrodes at the beginning and at the end of stimulation, making this condition indistinguishable from real (A-tDCS) stimulation. We did not counterbalance the order of sham and real stimulations across subjects (i.e., 5 day only A-tDCS, 5 days only S-tDCS) for two reasons: first, the number of patients (3) would have not allowed a complete balance of the stimulation order. Second, we intended to avoid potential carry-over effects if real stimulation had been applied first. In other words, hypothesizing that tDCS can produce enduring effects, the A-tDCS applied in the first 5 days could have produced unintended beneficial effects on the performances of the second period, when the S-tDCS was applied. We also decided to always start with S-tDCS in order to minimize the chances of short-term interference effects (Fertonani et al., 2010), given that 13 min of 1 mA tDCS significantly increases cortical excitability for up to 90 min after the end of stimulation (Nitsche and Paulus, 2000).

The two stimulations (sham and anodal) were administered 60 min apart (washing out interval).

Data were analyzed with SPSS 13.0 software (SPSS Inc., Chicago, IL, USA). Owing to the small sample size and preliminary nature of the study, we used the non-parametric McNemar chi-square test for repeated measures to compare the naming performances during S-tCDS and A-tDCS for the stimulation period in each patient. The same test was applied to compare the performances of the follow-up observations with the baseline level. A two-tailed *P*-value of 0.05 was used as a threshold for significance.

## Results

**Figure 3:**
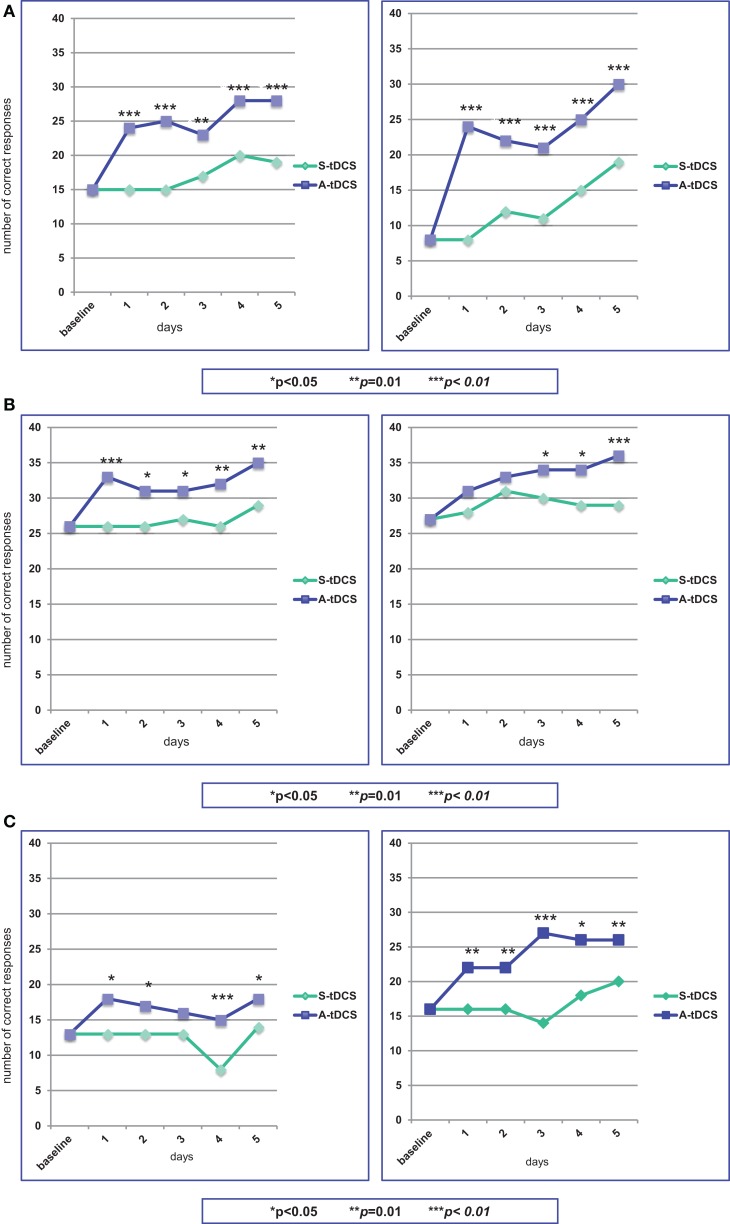
**Statistical analysis of the on-line effects of A-tDCS vs S-tDCS (McNemar test) on the first (left) and second (right) naming training**. **(A)** Patient #1; **(B)** Patient #2; **(C)** Patient #3.

The stimulation procedure was well tolerated and all the three patients were able to complete the study. The analysis showed that anodal stimulation caused a significant improvement in naming performance relative to baseline and sham condition for all three patients, the improvement being more pronounced for patient # 1. This was true for both sets of figures, indicating that difficulty in word frequency does not affect performance. Figures 3A–C provides a summary of the effects of A-tDCS and S-tDCS in each patient.

### Patient # 1

At baseline, he was able to name 15 out of 40 figures. During the first S-tDCS, his performance remained stable. During the first A-tDCS session, he named 24 figures (*p* = 0.003). In particular, he was able to name 7 out of 10 verbs as compared to baseline and S-tDCS (3 out of 10). In the following days of A-tDCS, the number of correct items raised up to 28, with an improvement of 47% as compared to S-tDCS (*p* = 0.003).

At the beginning of the second period of stimulation, patient #1 was able to name eight figures. This performance did not change during S-tDCS, while the A-tDCS increased the number of named figures to 24 (*p* = 0.0001). Low-frequency figures naming showed the best progress (from 1 to 11 items). Throughout this second A-tDCS stimulation period, the number of correctly named figures increased up to 30, with an improvement of 58% (*p* = 0.0009).

### Patient # 2

At baseline, patient # 2 could name 13 out of 40 figures, obtaining the same results during the first S-tDCS session. Concurrently with the first A-tDCS, he named 18 figures (*p* = 0.02). In the following days of A-tDCS, the number of correct responses did not notably change, remaining marginally, but significantly better than S-tDCS (*p* = 0.04).

Patient # 2 was able to name 16 of the second set of figures, both at baseline and during the first S-tDCS, while the number of correct items raised to 22 during the first A-tDCS (*p* = 0.014). The best performance was obtained for verb naming (from one to seven items). The number of named figures increased to 26 at the end of the second A-tDCS period of stimulation, with a 30% improvement (*p* = 0.014).

### Patient # 3

Patient #3 named 26 out of 40 figures at baseline and during the first S-tDCS session. The number of correct responses increased to 33 concurrently with the first A-tDCS (*p* = 0.008), particularly in low-frequency words (from 7 to 12 items). Performances during A-tDCS remained above 30 items for the entire period of stimulation, reaching 35 at the fifth day (*p* = 0.014).

A high number of correct responses characterized patient #3’s performance during the second period of stimulation. At baseline and during S-tDCS, 27 figures were correctly named. The first A-tDCS session caused an increase of named figures to 31, not reaching significance (*p* = 0.08). During the following days of A-tDCS, the number of correct responses progressively increased up to 36, becoming statistically significant (*p* = 0.008).

### Follow-up

**Figure 4:**
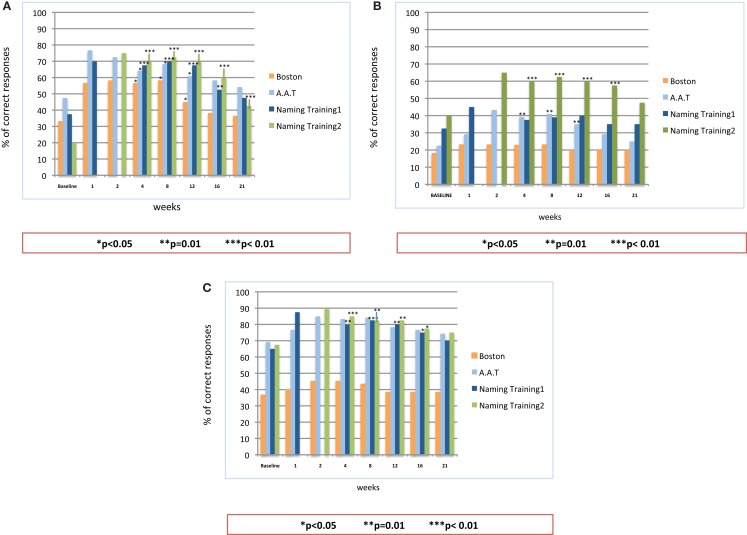
**Follow-up observation after the end of on-line A-tDCS: statistical analysis on the patients’ naming performance for naming list 1, naming list 2, Aachener Aphasie Test (AAT) and Boston Naming Test (BNT)**. **(A)** Patient #1; **(B)** Patient #2; **(C)** Patient #3.

All three patients were monitored and examined over a period of 21 weeks (5 months) after the end of the second stimulation period. Figure 4 provides the results of the follow-up examination.

The analysis showed a significant difference in the percentage of correct responses between the baseline and follow-up observations, but for patient #2, whose improvement reached the significance level only for the second naming training. For all the patients, the number of correct responses persisted significantly better than the baseline until the 16th week. Similarly to the stimulation period, patient #1 showed the best performance over time: as compared to baseline (37.5%), the percentage of correct responses at the 16th week was 52.5% with the first naming training (*p* = 0.01) and 60% after the stimulation with the second set of figures (*p* = 0.0001). The follow-up results of patient #2 showed an improvement in the percentage of correct responses for both sets of figures, though reaching significance only for the second one (*p* = 0.008 at the 16th week). For the first set of figures, the follow-up examination of patient #3 showed a significant improvement of the naming performances (*p* = 0.04 at the 16th week). The same levels of significance were obtained also for the second naming training. Up to the 21st week, the percentage of correct items for all the patients was still well above the baseline level. Such an improvement, however, did not reach the significance threshold, but for the performance of patient #1 after the training with the second list of figures (*p* < 0.005).

As to the standardized language tests AAT and BNT, all the patients obtained an enduring increment in the percentage of correct responses, as compared to the baseline. The level of significance, however, showed some inter-individual differences: patient #1 showed a significant improvement up to the 12th week (*p* < 0.05 for both AAT and BNT). This improvement slightly decreased over the following weeks, becoming not significant. Patient #2 obtained a significant improvement in the AAT up to the 12th week (*p* < 0.01), while the performances in the BNT, though improved, did not reach the significance level. As to patient #3, he achieved a better, but not significant, performance in both tests. Of note, for all the patients, both language test results persisted above the baseline level up to the 21st week.

## Discussion

Within the limits of a single-blind pilot study, our results in a small sample of subjects confirm previous reports of on-line A-tDCS efficacy in improving naming performances of post-stroke aphasic patients. We found a clear beneficial effect of A-tDCS (as compared to baseline and sham stimulation) in all our subjects. The importance of performing on-line A-tDCS (i.e., stimulation coupled with specific language training) in order to obtain significant naming improvement, repeatedly reported in the literature (Baker et al., 2010; Fridriksson et al., 2011; Marangolo et al., 2011), was also confirmed by our findings. The mild improvement of naming in the sham condition, which occurred only in some late sessions and never reached the significance level, was likely due to an after-effect induced by repeated A-tDCS (Monti et al., 2013).

The main result of our study, however, is that the beneficial effect of on-line A-tDCS was maintained over a period of up to 16 weeks after the end of stimulation. Of note, patients still showed a notable, though not significant, benefit up to the 21st week (5 months). To the best of our knowledge, the small pilot study here represents the first attempt at demonstrating a long-term beneficial effect of multiple sessions of tDCS on chronic post-stroke aphasia. The sustained improvement obtained in our patients suggests that tDCS is able to generate lasting changes in language outcome after stroke.

The beneficial effect of A-tDCS proved to be independent from word naming difficulty, both during the training sessions and in the follow-up period. The enhancement of recovery from language disturbances was not limited to the performances of naming training, but, to a lesser extent, it tended to generalize to untreated items (i.e., AAT and BNT denomination task). This raises the issue of generalization of improvement in restorative neurology (Thompson and Shapiro, 2007), also because previous studies on this topic reported conflicting results: Baker et al. (2010) reported a significantly improved naming accuracy for treated items that did not generalize to untreated items matched for complexity, while Marangolo et al. (2011) found an extension of the beneficial results on speech apraxia and speech production (Marangolo et al., 2013) to other language tasks. Further studies addressing this issue are required.

Surprisingly, tDCS studies on aphasic patients were so far conducted with a short, if any, follow-up period (Monti et al., 2013). The longest observation time was 3 weeks after the end of stimulation (Fridriksson et al., 2011), while Marangolo et al. (2011) were able to monitor their patients for 2 months. Their study, however, was aimed to verify tDCS efficacy in articulatory disorder of speech (apraxia of speech).

As suggested by some authors (Nitsche and Paulus, 2000; Nitsche et al., 2003), modifications of the synaptic connections of the NMDA receptors involved in long-term potentiation (LTP) are the likely source of the long-lasting beneficial effects of A-tDCS. Recently, tDCS has been reported to enhance brain-derived neurotrophic factor (BDNF) secretion and tyrosine receptor kinase B (TrkB) activation *in vitro* study, which means tDCS may promote language learning through promotion of synaptic plasticity (Fritsch et al., 2010). Consistent with our data are the findings by Kim et al. (2008), who reported two cases of chronic stroke patients whose neurological functions were improved by continuous cortical (epidural) stimulation associated with rehabilitation. The improvement in their patients persisted for 4 months. In a pilot, randomized controlled trial, Khedr et al. (2013) have recently shown that post-stroke motor recovery was enhanced and maintained by tDCS over a period of 3 months.

Our study has some limitations. First, besides deciding not to counterbalance the sessions across subjects, we applied S-tDCS always before A-tDCS. One consequence might be that the improvement for A-tDCS on each day of training could be due to the fact that our patients sufficiently practiced the naming procedure during the S-tDCS, thus being “warmed up” to the task. This, however, seems unlikely in our cases, since the two stimulations (sham and real) were separated by a 1 h pause interval. Of note, in all the three patients, for both the sets of pictures, a few items were correctly named during S-tDCS and not during A-tDCS, indicating a negligible effect of practice. Second, we used a single-blind approach with the pictures showed by the examiner. This could have created a potential interaction between the examiner (not blinded) and the patients. However, this work was performed as a pilot study, and requires confirmation by larger scale studies with double-blind paradigm. Caution is obviously needed to draw any firm conclusion on a study on just three patients. However, one could tentatively speculate that anodic stimulation, concurrent with behavioral intervention, might enhance the capacity for spared left hemispheric regions to make compensatory plastic changes promoting a durable improvement in the patients’ language skills. In addition, the long-lasting improvement observed in our patients might provide an important insight into the outcomes of long-term treatment of a chronic condition such as post-stroke aphasia.

It is tempting to hypothesize that A-tDCS, together with specific language training, repeated over a regular period of time (i.e., every 16 weeks, when the language performance starts to worsen) might maintain a higher, stable language performance in chronic aphasic patients over a very long period (months and years). Multiple, periodic maintenance sessions of on-line A-tDCS might be tailored to the single patient’s duration of naming performance. This kind of approach could be somehow similar (though theoretically different) to the periodic intramuscular injections of botulinum toxin that are usually used for the treatment of different types of chronic movement disorders.

Further studies on larger groups of aphasic patients would be necessary in order to test this intriguing hypothesis.

## Conflict of Interest Statement

The authors declare that the research was conducted in the absence of any commercial or financial relationships that could be construed as a potential conflict of interest.
